# Antidiarrheal Activity of 80% Methanolic Leaf Extract of* Justicia schimperiana*

**DOI:** 10.1155/2018/3037120

**Published:** 2018-02-06

**Authors:** Belay Mekonnen, Assefa Belay Asrie, Zewdu Birhanu Wubneh

**Affiliations:** ^1^Hospital Pharmacy, University of Gondar, Gondar, Ethiopia; ^2^Department of Pharmacology, College of Medicine and Health Sciences, University of Gondar, Gondar, Ethiopia

## Abstract

**Background:**

Diarrhea is one of the leading causes of preventable death in developing countries and mainly affects children and infants. It has been reported that the leaf of* Justicia schimperiana* is used as an antidiarrheal agent in Libo Kemekem district, northwest Ethiopia.

**Method:**

The 80% methanolic leaf extract of* J. schimperiana* was evaluated for its activity against castor oil-induced diarrhea, enteropooling, and gastrointestinal motility in mice.

**Results:**

Significant reduction (*p* < 0.001) in the total defecation and diarrheal drops was produced by all the test doses of the extract. Percentage inhibition of wet feces was 42.58, 65.07, and 74.96% at 100, 200, and 400 mg/kg doses of the extract, respectively. The extract also significantly inhibited castor oil-induced enteropooling at all test doses. The percent reduction in mean weight of intestinal contents was 66.96, 67.83, and 76.52% at 100, 200, and 400 mg/kg doses of the extract, respectively. The extract significantly reduced gastrointestinal movement of charcoal meal as well at 200 (*p* < 0.01) and 400 mg/kg (*p* < 0.001) doses.

**Conclusion:**

In conclusion, the methanolic leaf extract of* J. schimperiana* has an antidiarrheal activity and this supports the use of this plant in the treatment of diarrhea in the traditional settings.

## 1. Background

Diarrhea is the passage of three or more loose stools. It is characterized by increased gastrointestinal motility and secretion and a decrease in the absorption of fluid and electrolytes [1, 2]. Based on the duration, diarrhea is classified into 3 types: acute diarrhea (duration < 2 weeks), persistent diarrhea (duration from 2 to 4 weeks), and chronic diarrhea (duration of more than 4 weeks) [3]. Acute diarrhea is mainly caused by enteric pathogens including viruses, bacteria, and parasites whereas most cases of chronic diarrhea result from functional or inflammatory bowel disorders, malabsorption syndromes, and drugs [4, 5]. Pathogenic agents, such as* Cryptosporidium*,* Giardia lamblia*, and enteropathogenic bacteria, are thought to be the cause of persistent diarrhea [6].

Diarrheal disease is one of the leading causes of preventable death in developing countries, and it mainly affects children and infants [3]. According to the WHO and UNICEF reports, there are about 2.5 billion cases of diarrheal disease worldwide every year, and 1.9 million children below 5 years of age die from diarrhea each year, of whom most are from developing countries. Of all child deaths from diarrhea, 78% occur in African and Southeast Asian regions [7].

Many patients with a sudden onset of diarrhea have self-limited illness requiring no treatment. However, in severe cases, dehydration and electrolyte imbalance are the principal risks, particularly in infants, children, and elderly patients, thus requiring both nonpharmacologic treatments, such as oral rehydration therapy (ORT) and zinc supplements, and pharmacological treatments [8].

Antimotility and antisecretory agents are the mainstay in the treatment of diarrhea. Opioids and their derivatives continue to be widely used in the treatment of diarrhea. Diphenoxylate, difenoxin, and loperamide are commonly used opioid antidiarrheals. There are also many other drugs that have antimotility or antisecretory effects on the intestine and can be used for the treatment of diarrhea [9, 10]. Antimicrobial agents can reduce the severity and duration of infectious diarrhea [11]. Most of the enteropathogens which cause persistent diarrhea are treatable with antimicrobial therapy [12]. Generally, fluoroquinolones have become the drugs of choice for the empirical treatment of acute diarrhea in adults. On the other hand, third-generation cephalosporins have been considered the best drugs for the empirical treatment of severe acute infectious diarrhea in children [13].

Herbal medicines have been used for treating diarrheal diseases, and it is estimated that up to 80% of the population in developing countries depend on traditional medicines for primary healthcare [14]. There are an enormous number of herbal medicines around the world that are claimed to be effective in treating diarrhea [8].* Amaranthus caudatus, Coffea arabica, Balanites rotundifolia*,* Boscia coriacea*,* Cissampelos pareira*,* Plumbago zeylanica*,* Solanum hastifolium*,* Berberis crataegina*,* Cornus mas*,* Ecballium elaterium*,* Mentha longifolia, Rhamnus cathartica*, and* Teucrium polium *are some of those medicinal plants used in the treatment of diarrhea traditionally in different societies [15–17].

Medicinal plants are a promising source of new antidiarrheal drugs. For this reason, the WHO has encouraged studies pertaining to the treatment and prevention of diarrheal diseases using traditional medical practices [18]. Currently available drugs are linked with adverse effects and contraindications [19, 20]. Drug resistance is another challenge to think about antibiotics used in the treatment of diarrhea [21]. The high incidence of diarrhea in developing countries coupled with limitations of currently available antidiarrheal drugs and poor healthcare coverage may make traditional medicines good alternative agents for the management of diarrhea.

*Justicia schimperiana,* also known by synonyms* Adhatoda schimperiana *and* Gendarussa schimperiana,* has different local names in Ethiopia including “dhumuugaa” in Afaan Oromoo, “Sensel” or “Simiza” in Amharic, and “Surpa,” “Kasha,” or “Keteso” in Sidama. It belongs to the family of Acanthaceae. This plant has several medicinal uses in different areas of Ethiopia. Ethnobotanical study reports showed that the plant is used in the treatment of various ailments such as evil eye, hepatitis B (jaundice), rabies, asthma, common cold, stomachache, diarrhea, tapeworm infestation, anthrax, wound, external parasite, ascariasis, and skin irritation [15, 22–24].* Justicia schimperiana *is used in the treatment of malaria in Ethiopian folk medicine and experimental investigations confirmed that the crude extract of the plant showed an antimalarial activity [25]. Another experimental study also confirmed that chloroform, methanol, and aqueous fractions of the leaves of* Justicia schimperiana *had an antimalarial activity [26].* Justicia schimperiana *has been also traditionally used for the treatment of diabetes mellitus. One published experimental study determined that the aqueous leaf extract of the plant produced a fall in blood glucose level in normal and diabetic mice [27]. Furthermore, the phytochemical analysis on the* n*-hexane extract of* Justicia schimperiana* showed the presence of alkaloids, polyphenols, flavonoids, glycosides, saponins, triterpenes, and quinones [28].

It has been reported that* J. schimperiana *is traditionally used for the treatment of diarrhea in Libo Kemekem district, northwest Ethiopia. The leaves of the plant are smashed and mixed with water and then the juice is drunk for diarrhea [23].* J. schimperiana* has been used in the treatment of diarrhea without scientific proof for safety and efficacy. Investigating the safety and efficacy of this plant in an animal model could give valuable evidence of the claimed traditional use of the plant. Thus, the aim of this study was to evaluate the* in vivo *antidiarrheal activity of 80% methanolic leaf extract of* J. schimperiana *in mice.

## 2. Methods

### 2.1. Chemicals and Reagents

Distilled water (Ethiopian Pharmaceutical Manufacturing, Ethiopia), loperamide HCl (Bafna Pharmaceuticals, Ltd., India), castor oil (Shiba Pharmaceuticals and Chemicals, Yemen), methanol (Lab Chemicals, Avishkar, India), activated charcoal (Okhla Industrial Area, India), and atropine sulphate inj. (0.1%) (Jeil Pharm. Co. Ltd., Korea) were used. Other reagents, such as glacial acetic acid (Blulux Laboratories (P) Ltd., India), ammonia (Loba Chemie Pvt. Ltd., India), ferric chloride (Blulux Laboratories (P) Ltd., India), Mayer's reagent (Research-Lab Fine Chem Industries, India), hydrochloric acid (Blulux Laboratories (P) Ltd., India), chloroform (Nice Chemicals Pvt. Ltd., India), sulphuric acid (HiMedia Laboratories Pvt. Ltd., India), and benzene (Blulux Laboratories (P) Ltd., India), were used in the phytochemical screening test.

### 2.2. Collection of Plant Material

The fresh leaves of* J*.* schimperiana *were collected from a rural area around Gondar town called Blajig, northwest Ethiopia, in November 2015. Meanwhile, the fresh aerial part of the plant was sent to and authenticated at the National Herbarium of Ethiopia, Department of Plant Biology and Biodiversity Management, College of Natural Sciences, Addis Ababa University, Addis Ababa, Ethiopia. The plant specimen (voucher number BM/001) has been deposited there in the National Herbarium.

### 2.3. Preparation and Extraction of the Plant Material

The leaves of* J. schimperiana *were cleaned with distilled water and air-dried under shade at room temperature. Thereafter, the dried leaves were coarsely powdered using mortar and pestle. Then, 800 gm of powdered leaves was weighed using an electronic balance and extracted by maceration using 80% methanol for three consecutive days at room temperature with occasional stirring. After three days, the supernatant was filtered with Whatman filter paper No. 1. The residue was remacerated and filtered in the same way twice. The combined filtrates were concentrated in an oven set at 40°C. The dried extract was weighed and transferred into vials and kept in a desiccator in the laboratory at room temperature until the actual study procedures started [29].

Normally, in traditional medicine, people use the leaves of* J. schimperiana* for the treatment of diarrhea after smashing and mixing them with water as a juice, but here in this study we used 80% methanol extract of the plant because hydromethanolic solvents (especially 80% methanol) are usually better in extracting the most important chemical constituents of different plants [30, 31].

### 2.4. Experimental Animals

Swiss albino mice, weighing 18–30 g, 6 to 8 weeks of age, were used. The mice were bred at the Animal House of the Department of Pharmacology, University of Gondar, under standard conditions. They were housed in plastic cages with softwood shavings and chips as beddings, in a room with 12 : 12 dark-to-light cycle, at room temperature and 55% humidity, and with free access to clean water and pelletized food* ad libitum*. All mice were acclimatized to the working (lab.) environment one week prior to the experiment [32].

### 2.5. Grouping and Dosing of Animals

For each of the three antidiarrheal activity test models, 30 mice were used. The mice were randomly divided into five groups of six mice each for each model. In all models, the negative control groups were treated with the vehicle (distilled water, 10 ml/kg). The positive controls were treated with loperamide 3 mg/kg (in castor oil-induced diarrhea and enteropooling models) and atropine 1 mg/kg (in gastrointestinal motility test model). The other groups (Groups 3, 4, and 5) in each model received 100, 200, and 400 mg/kg doses of the crude extract, respectively.

### 2.6. Preliminary Phytochemical Screening

Qualitative phytochemical screening tests were carried out based on the methods used by Savithramma et al. and Jones and Kinghorn [33, 34] to determine the presence or absence of alkaloids, tannins, flavonoids, saponins, phenols, terpenoids, steroids, glycosides, and anthraquinones in the 80% methanolic leaf extract of* J. schimperiana.*

### 2.7. Acute Oral Toxicity Test

Acute oral toxicity test was conducted according to the OECD 425 guideline on five female Swiss albino mice at a single oral limit dose of 2000 mg/kg body weight [35]. Fife female Swiss albino mice were randomly selected for the test. One animal was first dosed at the limit test dose and survived in the 24-hour follow-up. Then, the other four additional animals were sequentially dosed at 2000 mg/kg so that a total of five animals were tested. The animals were observed individually for signs of toxicity at least once in the first 30 minutes after dosing, periodically during the first 24 hours, and daily for an additional 13 days, for a total of 14 days.

### 2.8. Castor Oil-Induced Diarrhea in Mice

This test was done based on the method used by Awouters et al. [36]. Thirty mice were fasted for 18 hours and divided into five groups with six animals in each group. The first group received distilled water (10 ml/kg) and the second group received loperamide (3 mg/kg), serving as negative and positive controls, respectively. Groups 3, 4, and 5 received 100, 200, and 400 mg/kg of the extract, respectively. After one hour, all the animals received 0.5 ml/animal of castor oil orally. The animals were kept in separate metabolic cages. The severity of diarrhea was assessed for 4 hours. The mean total number of feces (dry and wet diarrheal droppings) was determined and compared with the negative control group. The total score of diarrheal feces for the negative control group was considered as 100%. The percent inhibition of total defecation and that of diarrhea were calculated using the following formulas:

(1) %  inhibition  of  defecation=Total  number  of  feces  in  the  negative  control−Total  number  of  feces  in  treated  groupTotal  number  of  feces  in  the  negative  control×100,%  inhibition  of  diarrhea=Total  number  of  diarrheal  feces  in  the  negative  control−Total  number  of  diarrheal  feces  in  treated  groupTotal  number  of  diarrheal  feces  in  the  negative  control×100.

### 2.9. Castor Oil-Induced Enteropooling

The method employed by Chitme et al. [37] was used to determine the effect of the plant extract on castor oil-induced intestinal fluid accumulation. Thirty mice were deprived of food for 18 hours while water was allowed* ad libitum*. Then, the animals were grouped and treated in the same fashion as described for castor oil-induced diarrhea above. One hour later, 0.5 ml of castor oil was administered per mouse in all groups. The mice were sacrificed 1 hour after the administration of castor oil and the small intestine of each from the pylorus to the caecum was isolated and weighed. Then, the intestinal content of all individual animals was collected by milking into a graduated tube and the volume of each was measured. Just after removal of the intestinal content, the intestine of each mouse was reweighed. Then, percent reductions in the weight of intestinal content and volume of intestinal content, relative to the negative control group, were calculated using the following formulas:

(2) %  reduction  in  weight  of  intestinal  content=Weight  of  intestinal  contentgin  the  negative  control−Weight  of  intestinal  contentgin  treated  groupWeight  of  intestinal  contentgin  the  negative  control×100,%  reduction  in  volume  of  intestinal  content=Volume  of  intestinal  contentmlin  the  negative  control−Volume  of  intestinal  contentmlin  treated  groupVolume  of  intestinal  contentmlin  the  negative  control×100.

### 2.10. Gastrointestinal Motility Test

The mice were divided into five groups each containing six mice and fasted for 18 hours while water was provided* ad libitum*. The first group (control group) received distilled water orally (10 ml/kg), while the second group (positive control) received atropine (1 mg/kg IP). The mice in Groups 3, 4, and 5 were given 100, 200, and 400 mg/kg of the plant extract, respectively. After one hour, 0.5 ml of castor oil was given orally to each animal in all groups. Again, after an hour, 0.5 ml of 5% charcoal suspension in distilled water was given to each mouse orally. Then, all animals were sacrificed after 30 minutes and the small intestine of each mouse was isolated. The intestinal length moved by the charcoal meal from the pylorus towards the caecum was measured, and the percent of intestinal length travelled by the charcoal meal and percent inhibition of intestinal transit were determined as follows [38]:

(3) %  inhibition  of  intestinal  transit=%  intestinal  transit  of  charcoal  meal  in  the  negative  control−%  intestinal  transit  of  charcoal  meal  in  treated  group%  intestinal  transit  of  charcoal  meal  in  the  negative  control×100.

### 2.11. *In Vivo* Antidiarrheal Index (ADI)

*In vivo* antidiarrheal index (ADI) of treated groups was determined using data from castor oil-induced diarrhea, enteropooling, and gastrointestinal motility tests using the formula developed by Aye-Than as described below [39]:

(4) $$\begin{array}{c} ADI\;\;in\;\;vivo=\sqrt[{3}]{D\;\;freq\times G\;\;meq\times P\;\;freq}, \end{array}$$

where *D* freq is the delay in defecation time or diarrhea onset calculated as

(5) $$\begin{array}{c} D\;\;freq=\frac{mean\;\;onset\;\;of\;\;diarrhea\;\;in\;\;treated\;\;group-mean\;\;onset\;\;of\;\;diarrhea\;\;in\;\;the\;\;negative\;\;control\;\;group}{mean\;\;onset\;\;of\;\;diarrhea\;\;in\;\;the\;\;negative\;\;control\;\;group}\times 100, \end{array}$$

*G* meq is the charcoal meal travel reduction (as % of the negative control) from the gastrointestinal motility test model, and *P* freq is the reduction in the number of wet stools (as % of the negative control) from the castor oil-induced diarrhea model.

### 2.12. Statistical Analysis

Data was analyzed using SPSS statistical software, version 16. Results were expressed as means ± SEM. Comparisons between groups were made using ANOVA followed by post hoc Tukey's multiple comparison test. At 95% confidence interval (*p* < 0.05), the difference between the compared groups was considered as statistically significant.

### 2.13. Ethical Consideration

The experimental animals were handled and cared for during the experimental procedures according to the local ethical and internationally accepted laboratory animal use, care, and welfare guidelines [32]. The study was approved by the Experimental Animals Ethics Committee, Department of Pharmacology, University of Gondar, before the actual experimental activities were commenced.

## 3. Results

### 3.1. Preliminary Phytochemical Screening

The preliminary phytochemical screening test of 80% methanolic leaf extract of* J. schimperiana* showed the presence of phenols, tannins, flavonoids, saponins, terpenoids, glycosides, and anthraquinones.

### 3.2. Acute Oral Toxicity Test

In the oral acute toxicity test using the limit dose of 2000 mg/kg body weight, the 80% methanolic leaf extract of* J. schimperiana *was found to be safe as there was no mortality or remarkable signs of toxicity noted in the 14-day observation of the mice which were used for the test. This result confirmed that the LD50 of the plant extract is greater than 2000 mg/kg.

### 3.3. Effect of the Extract on Castor Oil-Induced Diarrhea

A significant reduction in the number of defecation instances was observed with all the test doses of the extract compared with the negative control group. The mean total number of defecation instances in extract-treated groups was 12 ± 0.93, 9.67 ± 0.88, and 5.50 ± 0.76 at 100, 200, and 400 mg/kg doses of the extract, respectively, while this value was 4.33 ± 0.49 in the loperamide-treated group. The percent inhibition of total defecation relative to the negative control group was 38.99, 50.84, and 72.04% at 100, 200, and 400 mg/kg doses of the extract, respectively. The standard drug had shown a more marked reduction in the number of defecation instances (77.99%) compared to the vehicle-treated group. The mean number of diarrheal drops in the extract-treated groups was 3.83 ± 0.60, 2.33 ± 0.42, and 1.67 ± 0.33 at 100, 200, and 400 mg/kg doses, respectively, while in the loperamide-treated group it was 1.33 ± 0.21. Percent inhibition of wet (diarrheal) feces was 42.58, 65.07, and 74.96% at the doses of 100, 200, and 400 mg/kg of the plant extract, respectively. The percent inhibition of diarrhea recorded for the loperamide-treated group was 80.06% (Table 1).

### 3.4. Effect of the Extract on Castor Oil-Induced Enteropooling in Mice

**Figure 1:**
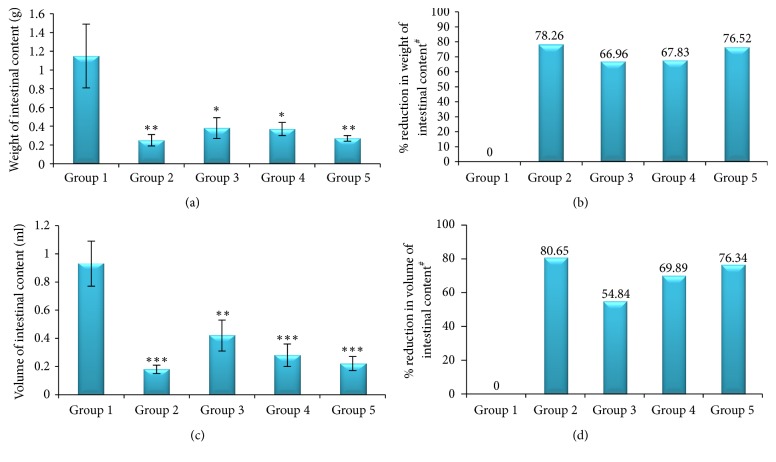
Effect of the methanolic leaf extract of* J. schimperiana* on castor oil-induced enteropooling in mice; (a) effect on the weight of intestinal content; (b) % reduction in the weight of intestinal content; (c) effect on the volume of intestinal content; (d) % reduction in the volume of intestinal content. Values expressed as mean ± SEM (*n* = 6); ^*∗*^*p* < 0.05; ^*∗∗*^*p* < 0.01; ^*∗∗∗*^*p* < 0.001 compared with the negative control; ^#^% reduction is relative to the negative control group.* Group 1*: mice that received distilled water (negative control);* Group 2*: mice treated with 3 mg/kg of loperamide (positive control);* Group 3*: mice treated with 100 mg/kg of the extract;* Group 4*: mice treated with 200 mg/kg of the extract;* Group 5*: mice treated with 400 mg/kg of the extract.

Compared to the negative controls, the three serial doses of* J. schimperiana *leaf extract significantly inhibited castor oil-induced enteropooling in mice at doses of 100, 200, and 400 mg/kg as revealed by reduction in weight and volume of intestinal contents. At 100, 200, and 400 mg/kg doses of the extract, the mean weights of the intestinal content were 0.38 ± 0.11, 0.37 ± 0.07, and 0.27 ± 0.03 g, while the mean volumes of the intestinal content were 0.42 ± 0.11, 0.28 ± 0.08, and 0.22 ± 0.05 ml, respectively. The highest dose of the plant extract (400 mg/kg) produced a more significant reduction in both the weight and the volume of the intestinal content. The plant extract decreased the mean weight of the intestinal content by 66.96, 67.83, and 76.52% and the volume of the intestinal content by 54.84, 69.89, and 76.34% at 100, 200, and 400 mg/kg doses, respectively, compared to the negative controls. The highest percent inhibition, 80.65%, was scored by loperamide (Figure 1).

### 3.5. Effect of the Extract on the Intestinal Transit of Charcoal Meal in Mice

**Figure 2:**
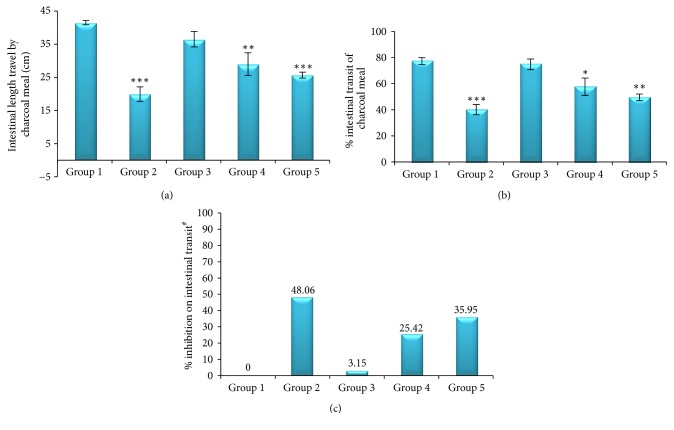
Effect of the methanolic leaf extract of* J. schimperiana* on the intestinal transit of charcoal meal in mice; (a) effect on intestinal transit; (b) effect on % intestinal transit; (c) % inhibition on intestinal transit. Values expressed as mean ± SEM (*n* = 6); ^*∗*^*p* < 0.05; ^*∗∗*^*p* < 0.01; ^*∗∗∗*^*p* < 0.001 compared with the negative control; ^#^% inhibition is relative to the negative control group.* Group 1*: mice that received distilled water (negative control);* Group 2*: mice treated with 1 mg/kg of atropine (positive control);* Group 3*: mice treated with 100 mg/kg of the extract;* Group 4*: mice treated with 200 mg/kg of the extract;* Group 5*: mice treated with 400 mg/kg of the extract.

The plant extract reduced castor oil-induced gastrointestinal movement of charcoal meal significantly at 200 (*p* < 0.01) and 400 mg/kg (*p* < 0.001) doses compared with the negative control in a dose-dependent manner. The highest effect was produced by 400 mg/kg dose of the extract, which was comparable to the effect of the standard drug. Significant reduction in % intestinal transit of charcoal meal was produced by 200 (57.58 ± 6.65%, *p* < 0.05) and 400 mg/kg (49.45 ± 2.54%, *p* < 0.01) and a more significant effect was produced by loperamide (40.10 ± 3.97%, *p* < 0.001), relative to the negative control group. Compared to the group that received distilled water, the percent inhibition of intestinal transit was 25.42 and 35.95% at 200 and 400 mg/kg doses of the plant extract, respectively. The highest percent inhibition of intestinal transit of charcoal meal, 48.06%, was produced by atropine (Figure 2).

### 3.6. *In Vivo* Antidiarrheal Index

The antidiarrheal index (ADI) is a measure of the combined value of different parameters used to evaluate the antidiarrheal activity of a given agent. The extract showed an antidiarrheal index of 13.88, 39.18, and 63.93 at doses of 100, 200, and 400 mg/kg, respectively, indicating a dose-dependent effect on the ADI value (Table 2).

## 4. Discussion

Several studies have validated traditionally used antidiarrheal plants by evaluating the effects of these plants on gastrointestinal transit and water and electrolyte secretion using animal models [40, 41]. Therefore, this study was proposed and conducted to evaluate the claimed antidiarrheal effect of* Justicia schimperiana *using antidiarrheal activity test models in mice.

As shown in Table 1, the plant extract significantly (*p* < 0.001) reduced the total fecal output and diarrheal drops in castor oil-treated mice in the four-hour observation compared with the negative controls. The three serial doses of the extract were found to produce a reduction in the frequency of defecation and diarrheal output to the same significant level that was produced by loperamide. Furthermore, at the highest dose, 400 mg/kg, the plant extract significantly (*p* < 0.001) delayed the onset of diarrhea caused by castor oil when compared with the negative controls. The percent reduction in the frequency of defecation and diarrhea was increased with a corresponding increase in the dose of the plant extract. The percent inhibition produced by the highest dose of the extract was closer to the inhibition produced by loperamide. This increasing pattern of percent inhibition in the total number of fecal output instances and diarrheal episodes with increasing dose of the extract plus significant delay in the onset of diarrhea by the highest dose implies that the plant extract inhibits diarrhea more effectively at relatively higher doses [42].

Similar to the findings in the castor oil-induced diarrhea model, all doses of the plant extract showed a significant reduction in both the mean weight and the volume of intestinal fluid compared with the negative controls. The percent reduction in weight and volume of intestinal content was increased with the dose of the extract. This result demonstrated that the effect of the plant extract on percent inhibition of castor oil-induced enteropooling is increased as its dose was increased. The results in this model revealed that the effect of the highest dose of the extract on enteropooling (intestinal fluid accumulation) was found to be closer to and comparable with the inhibitory effect of loperamide. The findings in this model may indicate that the plant extract has a significant antisecretory effect and this contributes to its antidiarrheal effect noted in castor oil-induced diarrhea model.

In the gastrointestinal motility test model (Figure 2), the 80% methanolic leaf extract of* J. schimperiana* reduced gastrointestinal motility in castor oil-treated mice as shown by reduction in gastrointestinal movement of charcoal meal. The extract caused a significant reduction in the gastrointestinal charcoal meal transit at 200 (*p* < 0.01) and 400 mg/kg (*p* < 0.001) doses compared with the negative controls. The effect of the smallest dose of the extract on this parameter was found to be insignificant. The percent inhibition in intestinal transit was 3.15, 25.42, and 35.95% at 100, 200, and 400 mg/kg doses of the extract, respectively. These findings indicate that the gastrointestinal motility was greatly decreased as the dose of the extract was increased. The reduction in gastrointestinal motility increases the time of stay of gastrointestinal contents in the intestine and this may promote intestinal water and electrolyte absorption. So, the antidiarrheal effect of the plant extract may be attributed to, at least in part, its antimotility activity.

In this study, the methanolic leaf extract of* J. schimperiana* has been shown to reduce castor oil-induced diarrheal episodes and intestinal secretion and motility. So, these results can be taken as scientific evidence showing that this plant has an antidiarrheal activity. Furthermore, the* in vivo* antidiarrheal index (ADI) of treated groups of mice, which relates delay in onset of diarrhea, percent inhibition of wet fecal output, and intestinal transit relative to the negative control group, indicates the dose-dependent effect of the plant extract [43]. But the exact mechanism of action of the plant extract for these effects was not determined. The possible mechanisms of action of the extract can be proposed based on the pathophysiologic processes of diarrhea and the actions of castor oil to induce diarrhea.

Diarrhea is caused by four pathophysiologic processes: increased luminal osmolarity, electrolytes secretion, decreased electrolytes absorption, and abnormal intestinal motility causing reduction in intestinal transit time [44]. In the intervention of diarrhea, antimotility and antisecretory agents remain as the main agents used to decrease such pathophysiologic changes [45]. Castor oil has been widely used for induction of diarrhea in antidiarrheal activity studies because it releases ricinoleic acid, a metabolite that causes diarrhea, upon metabolism in the gut [46]. Ricinoleic acid initiates diarrhea via mechanisms such as irritation of GI mucosa, leading to the release of prostaglandin which stimulates gastrointestinal motility and electrolyte secretion, reducing electrolyte absorption from the intestine and colon; these are similar to the pathophysiologic processes resulting in diarrhea [47]. Therefore, the antidiarrheal activity of the plant might be due to the activities that oppose the actions of castor oil for induction of diarrhea or pathophysiologic processes leading to diarrhea. The extract has been shown to decrease the intestinal fluid accumulation. This suggests that the plant extract may decrease water and electrolyte secretion to the intestinal lumen while promoting their absorption, which in turn could decrease intestinal overload and distension, leading to a decrease in intestinal motility (giving a longer time for absorption) and water contents of the fecal drops and hence overall reduction in the total number of defecation instances and diarrheal drops in treated groups. This is consistent with the mechanism of action of loperamide for its antidiarrheal effect as presented in the literatures [48]. In addition, the extract may have an anticholinergic activity and cause reduction in intestinal motility and secretion, which is in agreement with the action of atropine on the intestine [49].

The methanolic leaf extract of* J. schimperiana* contains phenols, tannins, flavonoids, saponins, terpenoids, glycosides, and anthraquinones as asserted by preliminary phytochemical screening tests, and most of these secondary metabolites were reported to have an antidiarrheal activity. Reports in the literatures showed that tannins have an antispasmodic and muscle relaxant effect, flavonoids inhibit prostaglandin E_2_-induced intestinal secretion, saponins inhibit histamine release, terpenoids inhibit the release of prostaglandins, and phenols reduce intestinal secretion and transit and have an astringent action. All these actions lead to the inhibition of diarrhea by decreasing intestinal secretion and motility [50–52]. Therefore, the antidiarrheal activity of the plant extract may be produced by these chemical constituents. Moreover, the plant extract was found to be safe as no sign of toxicity was noted in the acute oral toxicity test. This indicates that the plant is tolerable and safe even at higher doses than used in the three antidiarrheal models of this study. This validates the safety of the plant in its use in the traditional settings as well.

## 5. Conclusion

In conclusion, the methanolic leaf extract of* J. schimperiana *has an antidiarrheal activity as revealed by reductions in the total fecal output and diarrheal drops, intestinal fluid accumulation, and gastrointestinal motility. Hence, this study supports the use of the plant in the treatment of diarrhea in the traditional settings.

## Figures and Tables

**Table 1:** Effect of methanolic leaf extract of *J. schimperiana* on castor oil-induced diarrhea in mice.

Group	Dose (mg/kg)	Onset of diarrhea (min)	Total number of feces	% inhibition of defecation^#^	Weight of stool (g)	Total number of diarrheal feces	% inhibition of diarrhea^#^
Group 1 (negative control)	-	66 ± 1.48	19.67 ± 0.88	-	0.60 ± 0.11	6.67 ± 0.88	-
Group 2 (positive control)	3	202 ± 6.25^*∗∗∗*^	4.33 ± 0.49^*∗∗∗*^	77.99	0.24 ± 0.05	1.33 ± 0.21^*∗∗∗*^	80.06
Group 3 (extract)	100	79.17 ± 2.30	12.00 ± 0.93^*∗∗∗*^	38.99	0.54 ± 0.12	3.83 ± 0.60^*∗∗∗*^	42.58
Group 4 (extract	200	90.00 ± 7.64	9.67 ± 0.88^*∗∗∗*^	50.84	0.32 ± 0.09	2.33 ± 0.42^*∗∗∗*^	65.07
Group 5 (extract)	400	130.00 ± 8.16^*∗∗∗*^	5.50 ± 0.76^*∗∗∗*^	72.04	0.23 ± 0.07	1.67 ± 0.33^*∗∗∗*^	74.96

Values expressed as mean ± SEM (*n* = 6); ^*∗∗∗*^*p* < 0.001 compared with the negative control; ^#^% inhibition is relative to the negative control group. Negative control: a group of mice that received distilled water. Positive control: a group of mice treated with loperamide.

**Table 2:** *In vivo* antidiarrheal index of the methanolic leaf extract of *J. schimperiana*.

Group	Dose (mg/kg)	Delay in diarrhea onset (*D* freq)	Charcoal meal travel reduction (*G* meq)	Reduction in the number of wet stools (*P* freq)	*In vivo* ADI
Group 1 (negative control)	-	-	-	-	-
Group 2 (positive control)	-	206.06	48.06	80.06	92.55
Group 3 (extract)	100	19.95	3.15	42.58	13.88
Group 4 (extract	200	36.36	25.42	65.07	39.18
Group 5 (extract)	400	96.97	35.95	74.96	63.93

Negative control: a group of mice that received distilled water. Positive control: a group of mice treated with loperamide (3 mg/kg) in a castor oil-induced diarrhea model and atropine (1 mg/kg) in a gastrointestinal motility test model.
